# When Hepatitis Masks Visceral Leishmaniasis: A Case of Secondary HLH in a Low‐Endemic Region

**DOI:** 10.1002/ccr3.71351

**Published:** 2025-10-23

**Authors:** Chernet T. Mengistie, Biruk T. Mengistie, Temesgen H. Gebremeskel, Thomas M. Muluneh, Rediet G. Kebede

**Affiliations:** ^1^ School of Medicine, College of Health Sciences Addis Ababa University Addis Ababa Ethiopia

**Keywords:** hemophagocytic lymphohistiocytosis, hepatitis‐like presentation, liposomal amphotericin B, secondary HLH, visceral leishmaniasis

## Abstract

Visceral leishmaniasis (VL)–associated hemophagocytic lymphohistiocytosis (HLH) is a rare but life‐threatening hyperinflammatory syndrome. Diagnosis is often challenging due to overlapping clinical features, especially in low‐endemic regions, where VL is not initially suspected. Severe hepatic dysfunction can further obscure the diagnosis and complicate treatment. We report a 14‐year‐old male from West Gojjam, Ethiopia, presenting initially with jaundice and clinical features of acute hepatitis. Over 3 months, he developed progressive abdominal distension, fever, pancytopenia, and coagulopathy. Imaging showed hepatosplenomegaly and ascites; viral and autoimmune hepatitis were excluded. Bone marrow examination revealed *Leishmania donovani* amastigotes and hemophagocytosis, confirming VL‐triggered secondary HLH. Due to severe hepatic impairment, etoposide was withheld. The patient was treated with intravenous dexamethasone and a six‐day course of liposomal amphotericin B, along with supportive care. Clinical and laboratory parameters improved steadily, with full recovery by 4 weeks. VL‐HLH should be considered in patients with hepatitis‐like presentations, even in low‐endemic areas. Early recognition and treatment targeting the underlying infection with amphotericin B plus corticosteroids can achieve favorable outcomes, even when etoposide is contraindicated.

**Summary**

- Visceral leishmaniasis‐associated hemophagocytic lymphohistiocytosis (VL‐HLH) can present with severe hepatitis‐like symptoms, especially in low‐endemic regions, complicating diagnosis.
- Early recognition and treatment with liposomal amphotericin B and corticosteroids, even without etoposide, can lead to full recovery, highlighting the need for tailored management in patients with hepatic dysfunction.

## 1 Introduction

Hemophagocytic lymphohistiocytosis (HLH) is a severe hyperinflammatory syndrome caused by uncontrolled activation of lymphocytes and macrophages, resulting in a cytokine storm and multiorgan failure [1, 2]. It may be primary (genetic) or secondary to triggers like infection, malignancy, or rheumatologic disease [1]. Untreated HLH is often fatal; mortality rates range ~8%–22% despite therapy [3]. The current standard treatment (HLH‐94 protocol) combines immunosuppressive chemotherapy (etoposide) and steroids, which dramatically improve survival in primary HLH [4], but high mortality persists without rapid intervention.

Visceral leishmaniasis (VL) is a protozoan disease caused by *Leishmania* spp., transmitted by sandflies. It is endemic in parts of Asia, Africa, and Latin America [5]. Globally, tens of thousands of VL cases occur annually, with ~13,000 reported in 2020 [6]. VL classically causes prolonged fever, weight loss, and massive hepatosplenomegaly with anemia or pancytopenia [5, 7]. In children, VL is more common due to less acquired immunity [5]. Although liver involvement and elevated transaminases occur, profound hepatitis is unusual; few cases of VL presenting as hepatitis have been described [8]. Liposomal amphotericin B is a first‐line therapy for VL in many settings [5].

Infections are a leading cause of secondary HLH, and among protozoa, *Leishmania* is the most common trigger [9, 10]. Visceral leishmaniasis–associated HLH (VL–HLH) is rare in children [11]. Published pediatric VL–HLH cases have reported a mortality rate as high as 100% if diagnosis and treatment are delayed [10], though studies confirm that prompt recognition and anti‐leishmanial therapy lead to good outcomes [11, 12]. However, in nonendemic areas, VL may not be initially suspected, especially when patients present with unusual features such as marked hepatitis or ascites. VL–HLH can be mistaken for other diseases (e.g., viral hepatitis or autoimmune liver disease) [8]. This report describes a child from a low‐endemic region who presented with HLH and prominent hepatic inflammation. His case underscores the need to consider VL in the differential diagnosis of pediatric HLH, even when hepatic involvement “masks” the parasitic infection.

## 2 Clinical History/ Examination

A 14‐year‐old male from West Gojjam, Amhara region of Ethiopia, an area without well‐documented local transmission of visceral leishmaniasis (VL), presented initially with progressive jaundice, fatigue, and right upper quadrant discomfort. He had no history of travel to recognized visceral leishmaniasis–endemic areas, such as Metema or Humera. He had lived in the same rural community since birth. Over 3 months, he developed progressive abdominal distension, easy fatigability, intermittent high‐grade fever, and worsening jaundice.

On admission, he appeared acutely ill with marked pallor, icteric sclera, and periorbital puffiness. His vital signs were: temperature 39.2°C, pulse rate 116 beats/min, respiratory rate 22 breaths/min, and blood pressure 100/60 mmHg. Abdominal examination revealed distension with ascites and a markedly enlarged spleen palpable 10 cm below the left costal margin. Grade 2 pitting edema was noted in both lower limbs. Neurological examination was normal, with preserved mental status.

## 3 Differential Diagnosis, Investigations, and Treatment

Imaging studies included abdominopelvic ultrasound, which confirmed hepatomegaly, splenomegaly, and ascites. Chest X‐ray was unremarkable. Initial laboratory investigations showed pancytopenia with a white blood cell count of 0.5 × 10^9^/L (neutrophils 30%, lymphocytes 62%, monocytes 3%), hemoglobin 5.4 g/dL, and platelet count 65 × 10^9^/L. Liver enzymes were markedly elevated (AST 270 U/L, ALT 315 U/L), and total bilirubin was 16 mg/dL with hypoalbuminemia (2 g/dL) and prolonged INR (2.08). Serum ferritin was 3000 ng/mL, and triglycerides were 492 mg/dL, consistent with hyperinflammation and hepatic dysfunction (Table 1).

**TABLE 1:** Laboratory findings at admission.

Parameter	Result	Reference range
Hemoglobin	5.4 g/dL (nadir)	12–16 g/dL
WBC count	0.5 × 10^9^/L	4–10 × 10^9^/L
Neutrophils	30%	—
Lymphocytes	62%	—
Monocytes	3%	—
Platelet count	65 × 10^9^/L	150–450 × 10^9^/L
AST (aspartate aminotransferase)	270 U/L	0–35 U/L
ALT (alanine aminotransferase)	315 U/L	0–35 U/L
Total bilirubin	16 mg/dL	0.1–1.2 mg/dL
Serum albumin	2 g/dL	3.5–5 g/dL
INR	2.08	0.8–1.2
Ferritin	3000 ng/mL	30–400 ng/mL
Triglycerides	492 mg/dL	< 150 mg/dL
Fibribogen	310 mg/dL	200–400 mg/dL
HBsAg	Negative	—
Anti‐HCV	Negative	—
Anti–HAV IgM	Negative	—
Antinuclear antibody	Negative	—
Anti–smooth muscle antibody	Negative	—

Coagulopathy was present, necessitating plasma and vitamin K administration. Screening for viral hepatitis (hepatitis B surface antigen, anti‐HCV antibodies), HIV, malaria antigen, and autoimmune hepatitis (antinuclear antibodies) was negative.

Given the pancytopenia, hyperferritinemia, and hypertriglyceridemia, a bone marrow aspirate was performed, revealing trilineage hematopoiesis with numerous macrophages actively engulfing erythroid and myeloid precursors (hemophagocytosis) and intracellular amastigotes of *Leishmania donovani*. A rapid rK39 serologic test was strongly positive, confirming visceral leishmaniasis. Due to resource constraints, NK‐cell activity and soluble CD25 levels were not assessed.

Secondary hemophagocytic lymphohistiocytosis (HLH) triggered by VL was diagnosed. Considering the severe hepatic impairment, etoposide therapy was deferred. The patient was treated with high‐dose intravenous dexamethasone (10 mg/m^2^/day for the first 2 weeks, followed by a gradual taper over 4 weeks) and a 6‐day course of liposomal amphotericin B (3 mg/kg/day). Supportive care included packed red cell transfusions and fresh frozen plasma. The duration of corticosteroid treatment was determined by the patient's clinical improvement and normalization of inflammatory markers.

## 4 Outcome and Follow‐Up

The patient demonstrated marked clinical and laboratory improvement over 2 weeks, with resolution of fever, jaundice, and abdominal distension. At discharge, his white blood cell count increased to 1 × 10^9^/L, hemoglobin to 9.9 g/dL, and coagulation parameters normalized. At four‐week follow‐up, he remained afebrile with normalized blood counts and mild residual hepatosplenomegaly.

## 5 Discussion

This case highlights the diagnostic complexity of hemophagocytic lymphohistiocytosis (HLH) triggered by visceral leishmaniasis (VL) in a region with low endemicity. Although Ethiopia is recognized as an endemic country for visceral leishmaniasis, the distribution of cases is geographically variable. High‐burden foci are concentrated in the lowland areas of northwest and southwest Ethiopia, while cases from other zones, including parts of Amhara, are less frequently reported [13]. The principal vector, *Phlebotomus orientalis*, has been identified in northwestern Ethiopia, particularly in Acacia–Balanites forests, and suitable habitats extend into neighboring districts of Amhara [14]. The occurrence of VL‐HLH in our patient, who had not traveled to established foci, raises the possibility of underrecognized local transmission. The patient initially presented with features resembling acute hepatitis, which delayed consideration of VL‐HLH.

Secondary HLH triggered by infections, including protozoan pathogens, poses significant diagnostic and therapeutic challenges in pediatric patients [9, 10]. While VL‐HLH is a recognized complication, it is uncommon in children, particularly outside endemic regions. In a recent systematic review, only 135 pediatric VL‐HLH cases were identified in the literature [11]. Studies from endemic areas (India, Africa, Latin America) account for most reports. In Europe and other low‐endemic regions, sporadic pediatric cases occur (often in immigrants or travelers) [5, 15].

Diagnosing VL‐HLH is challenging because both conditions share features, including fever, hepatosplenomegaly, cytopenias, and elevated ferritin/triglycerides [1, 9, 11]. In our patient, the prominent liver enzyme elevation and ascites raised concern for viral or autoimmune hepatitis, which delayed considering VL. This is consistent with prior reports showing that *Leishmania* infection can cause severe hepatic inflammation and granuloma formation, potentially “masking” the diagnosis [8]. Important clues that may help differentiate VL‐HLH include a history of travel to or residence in endemic areas and a lack of response to conventional treatments [12]. One study found that HLH secondary to VL was associated with higher inflammatory markers such as CRP and monocytopenia compared to uncomplicated VL [16]. However, no single finding is pathognomonic. Bone marrow aspiration remains a key diagnostic tool: Leishmania amastigotes were seen in 85% of cases in one review, although up to 38% required repeat testing for detection [11]. Rapid serologic tests like rK39 are sensitive in endemic settings, but can yield false negatives [17]. When routine tests fail, advanced methods such as metagenomic next‐generation sequencing (mNGS) have proven useful in detecting VL in HLH cases [10].

The diagnosis of hemophagocytic lymphohistiocytosis (HLH) was established based on the HLH‐2004 criteria, which require five of eight features: fever, splenomegaly, cytopenias, hypertriglyceridemia and/or hypofibrinogenemia, hemophagocytosis in bone marrow or other tissues, low or absent NK‐cell activity, hyperferritinemia, and elevated soluble CD25. In our patient, fever, splenomegaly, pancytopenia, hypertriglyceridemia, hypofibrinogenemia, elevated ferritin, and bone marrow hemophagocytosis fulfilled these criteria [18, 19].

Effective therapy for VL‐HLH must address both the infection and hyperinflammation. However, many experts emphasize that control of the underlying infection is critical. In fact, multiple pediatric series and reviews report that treating VL alone (often with amphotericin) frequently suffices to reverse HLH, without needing full HLH chemotherapy [12, 18]. For example, Mottaghipisheh et al. found that nearly all VL‐HLH patients in Iran recovered with anti‐leishmanial therapy alone [12]. Similarly, a Brazilian series recommended starting liposomal amphotericin promptly and reserving steroids or etoposide only for inadequate responders [18]. In our case, the patient recovered fully with amphotericin B and corticosteroids, as etoposide was withheld due to severe hepatitis. While etoposide remains a key component of HLH protocols, its use in infection‐associated HLH, particularly non‐EBV forms, is considered optional and should be individualized. Current guidance supports reserving etoposide for cases unresponsive to appropriate anti‐infective therapy or with progressive organ dysfunction, rather than routine use in all secondary HLH presentations [20].

Outcomes in VL‐HLH are generally good if diagnosed early. The systematic review found only 1.5% mortality among treated children [11]. By contrast, historical VL‐HLH series reported very high fatality rates without timely therapy [10]. Our patient's uneventful recovery is consistent with these data. Pediatric HLH mortality overall (all causes) is 8%–22%, but secondary HLH from treatable infections tends to fare better [3]. Indeed, Mottaghipisheh et al. reported 100% 3‐year survival in VL‐HLH [12]. Relapses of VL‐HLH are uncommon after complete amphotericin courses. In a large European pediatric VL cohort, relapses and adverse events were each ≈3% [16], reflecting the effectiveness of current regimens.

The favorable outcome in this case underscores the importance of early recognition and targeted therapy for VL‐HLH, even in resource‐limited settings where access to full diagnostic and therapeutic options is constrained. Prompt initiation of liposomal amphotericin B and corticosteroids led to full recovery, demonstrating that timely, evidence‐based management can achieve good outcomes even when etoposide or advanced immunological tests are unavailable.

## 6 Conclusion

Visceral leishmaniasis–associated HLH is a rare but life‐threatening condition that can mimic severe hepatitis, particularly in regions of low endemicity. This case underscores the importance of maintaining a high index of suspicion for VL‐HLH in patients with unexplained fever, hepatosplenomegaly, and pancytopenia, regardless of geographic setting. Our patient's recovery without etoposide, due to hepatic dysfunction, highlights that early recognition and targeted anti‐leishmanial therapy combined with corticosteroids can be life‐saving even in resource‐limited environments.

## Author Contributions

**Chernet T. Mengistie:** conceptualization, visualization, writing – original draft. **Biruk T. Mengistie:** visualization, writing – original draft, writing – review and editing. **Temesgen H. Gebremeskel:** resources, writing – review and editing. **Thomas M. Muluneh:** data curation, resources. **Rediet G. Kebede:** data curation, writing – review and editing.

## Ethics Statement

IRB review and approval was waived for this case report.

## Consent

Written informed consent was obtained from the patient for publication of the case details and accompanying images.

## Conflicts of Interest

The authors declare no conflicts of interest.

## Data Availability

The data that support the findings of this study are available from the corresponding author upon reasonable request.
